# Linking social–emotional competence, learning engagement, and teacher–student relationship to academic achievement: a structural equation model approach

**DOI:** 10.3389/fpsyg.2026.1756766

**Published:** 2026-03-16

**Authors:** Hanxue Li, Shuai Ma, Yan Liu

**Affiliations:** 1 School of Elementary Education, Hunan First Normal University, Changsha, China; 2 Department of Agricultural Leadership, Education and Communications, Texas A&M University, College Station, TX, United States

**Keywords:** academic achievement, learning engagement, pre-service teachers, social construction theory, social–emotional competence, teacher–student relationship

## Abstract

**Introduction:**

Relationships play an essential role in learning, particularly in peer relationships and the teacher–student relationship. Social–emotional competence, learning engagement, and teacher–student relationship can directly influence students’ academic achievement.

**Methods:**

This study examines the relationship among these fac¬tors and explores how they collectively link to the engagement and academic achievement of 1,528 pre-service teachers in a normal university through Social Construction Theory. We analyzed the data using the Structural Equation Modeling (SEM).

**Results:**

Findings revealed that all dimensions of social–emotional competence (self-relationship, interpersonal relationship, collective relationship, and responsible decision making) and the teacher–student relationship are associated with learning engagement. Two dimensions of social–emotional competence, collective relationship and responsible decision-making, are significantly correlated to academic achievement.

**Discussion:**

This study offers guidance and recommendations for pre-service teachers to enhance their overall growth in engagement and learning outcomes.

## 1 Introduction

The students’ academic achievement, an essential indicator to measure the quality of higher education, not only relates to the future development of students themselves, but also has a profound impact on the technology innovation and sustainable development of society (Idoiaga Mondragon et al., 2023). However, with the rapid development of the knowledge economy and the iterative progress of information technology, the educational environment has become more complex. Consequently, the factors affecting the academic achievement of university students have become more diverse. Relationships play an essential role in learning, particularly in both teacher–student and peer to peer relationships. This is especially evident for pre-service teachers, as they need to be fully prepared to handle all kinds of relationships including student teacher relationships in the future. Social–emotional competence (SEC), which includes self-relationship, collective relationship, interpersonal relationship and responsible decision-making, is vital for pre-service teachers (Chen et al., 2023). Therefore, pre-service teachers with good social–emotional competencies (SECs) can recognize and manage the triple relationships with themselves, others, and the collective groups, as well as the abilities to make responsible decisions.

According to the previous research, SECs not only influence the students’ learning motivation, learning attitude (Kawtharani and Halwani, 2020), and interpersonal relationship circle (Li et al., 2024), but also significantly affect their academic performance or learning outcomes (Kawtharani and Halwani, 2020; Rhoades et al., 2011; Wirajaya et al., 2019). SECs have also been confirmed as a vital predictor of students’ engagement (Santos et al., 2023). In addition, learners’ performance or achievement is largely determined by the extent of their learning engagement (Afzal and Crawford, 2022; Liu et al., 2023; Peng et al., 2017). Learning engagement is influenced by many factors including teacher–student relationship. It is a significant component of the education and the most basic and important interpersonal relations in education that has a non-negligible influence on students’ learning outcomes or academic achievement (Eleje et al., 2022; Munir et al., 2021). A positive teacher–student relationship can stimulate students’ interest in learning and promote their learning engagement with emotional support and learning guidance (Van Herpen et al., 2024).

Social–emotional competencies, learning engagement, and teacher–student relationships have gained significant attention in education research. Those factors are important predictors of students’ academic achievement. Previous studies have explored the correlation/associations between SEC, learning engagement, teacher–student relationship, and academic achievement. However, few studies explore how the various components of SECs relate to pre-service teachers’ academic achievement, nor the collective influence of these variables. Moreover, little research has investigated the influence mechanism of these variables on students’ academic achievement from the perspective of Social Construction Theory.

With the continuous updates to educational technology and rapid changes to the educational environment, the learning needs and learning styles of pre-service teachers are also undergoing profound changes, which makes it of great practical significance to re-examine the influence of these variables on academic achievement, and how they interact with each other. Therefore, this study aims to explore how the specifics of SECs, learning engagement, and teacher–student relationships are associated with engagement and pre-service teachers’ academic achievement. It seeks to reveal the internal connection and path among these variables. We aim to provide insights for educational administrative leaders, universities, and teachers to better understand the learning needs and characteristics of pre-service teachers to optimize education and instruction strategies.

## 2 Literature review

### 2.1 Learning engagement and academic achievement

Learning engagement is essential to the learners’ academic achievement (Liu et al., 2024). For instance, Afzal and Crawford (2022) found that student engagement is closely related to their academic achievement in online learning environment. A meta-analysis study conducted by Doo and Kim (2024) has found that learning engagement is a strong indicator of learning outcomes in online learning in higher education, but learning engagement showed a small-to-medium effect size. Liu et al. (2023) found a positive correlation between student engagement and English achievement of non-English majors in China. Additionally, Hsieh and Yu (2023) found the Individual-Oriented Achievement Motivation (IOAM) had a significant relationship to practical STEM competence gained via active participation and interaction with instructors. Besides, some studies neither take into account specific subjects of learning nor specific learning environments or contexts. According to Mekvabidze (2015), there is a direct causal relationship between students’ motivation, students’ engagement in the study process, and student achievement. Furthermore, it indicates that students’ engagement positively relates to their learning outcome, while a specific type of engagement is linked to a particular learning outcome, which means that not every engagement type has an equal impact thereon (Peng et al., 2017). In summary, learning engagement is a positive predictor for students’ academic achievement.

### 2.2 Social–emotional competence and academic achievement

There is different categorization of social–emotional competence. One of the famous frameworks of social–emotional competencies (SECs) is CASEL, which includes five broad and interrelated areas of competence: self-awareness, self-management, social awareness, relationship skills, and responsible decision-making [Collaborative for Academic Social and Emotional Learning (CASEL), 2003]. Domitrovich et al. (2017) divided social–emotional competence into two major fields: intrapersonal and interpersonal competencies. Chinese scholars have classified SEC into four dimensions based on the characteristics of Chinese students: self-relationship, collective relationship, interpersonal relationship, and responsible decision-making (Chen et al., 2023). The benefits of social–emotional competence for students’ development are evident. For instance, social–emotional competence is critical for healthy development and for counteracting the negative effects of exposure to risk (Domitrovich et al., 2017). A cross-lagged analysis found that social–emotional competence (SEC) is a key factor in the prevention of school bullying (Yang et al., 2025).

Moreover, social–emotional competence is generally regarded as an important factor promoting students’ academic achievement. For instance, young children’s social–emotional competence is associated with later academic success (Rhoades et al., 2011). There are also relations between seventh graders’ social–emotional competencies and their English academic achievement in Indonesia (Wirajaya et al., 2019). Zahid et al. (2024) revealed a low positive relationship between SEC and academic achievement. In addition, a gender-based study explored which skills of social–emotional competence affect their academic performance. The results showed that a higher level of emotional skills resulted in better grades for girls with no difference in self-management. Boys have stronger self-management skills, while girls have stronger relationship management skills (Portela-Pino et al., 2021).

### 2.3 Social–emotional competence and learning engagement

Social–emotional competence and learning engagement positively impact students’ academic achievement and development. SEC is closely related to learning engagement. A systematic review conducted by Santos et al. (2023) found that SECs are positively associated with students’ engagement, with similar results found at the middle school, high school, and university level with students from different backgrounds. The influence of SECs on students’ engagement is mediated through the teacher–student relationship, student–student relationship, and school identification (Yang, 2025). Meanwhile, a small number of studies explored the relationship between students’ specific SECs and their mental characteristics. For instance, collective relationship competence negatively predicts psychological anxiety based on the sample of 70,678 students from 101 Chinese universities (Chen and Chen, 2025). González-Sanz and Pichardo (2018) found a significant correlation between emotional competencies and responsible decision-making in pre-adolescents.

However, few studies examined how specific SECs (self-relationship skills, interpersonal relationship skills, collective relationship skills, and responsible decision-making) are related to academic achievement or learning engagement. Therefore, it is unclear which specific SECs affect students’ academic performance and learning engagement.

### 2.4 Teacher–student relationship and academic achievement

Teacher–student relationship is the core bond of educational activities, which runs through the entire education process and has a profound influence on students’ academic development and learning outcomes regardless of higher education or primary and secondary education. For instance, the teacher–student relationship has a significant impact on the learning outcome of middle school students (Eleje et al., 2022). Similarly, teachers’ relationship with students to a high extent influences the learning outcome of high school students (Obilor, 2019). Teacher–student interaction is highly correlated with college and university students’ academic achievement (González and Paoloni, 2015; Munir et al., 2021). To sum up, the teacher–student relationship is a relevant predictor of university learning behaviors or outcomes.

### 2.5 Teacher–student relationship and learning engagement

The previous research has confirmed that the teacher–student relationship is largely positively correlated with learning engagement. For instance, a positive teacher–student relationship positively predicts student engagement, and peer relationships also partially mediate this relationship (Wang and Yuan, 2024). It indicated that the perceived teacher–student relationship had a direct impact on student engagement among Chinese English learners (Li, 2023). A meta-analysis revealed that the teacher–student relationship positively influences the students’ learning engagement (Li and Xue, 2023). Moreover, it reported that there are significant gender differences in the correlation between teacher–student relationship and learning engagement among elementary students (Kang et al., 2023). Furthermore, He and Li (2024) found that student engagement plays a mediating role in the teacher–student relationship and students’ creativity. In summary, the teacher–student relationship directly or indirectly positively predicts learning engagement. Therefore, we can infer that SEC, student engagement, and teacher–student relationship are all relevant predictors of university students’ academic achievement.

## 3 Theoretical framework

**Figure 1:**
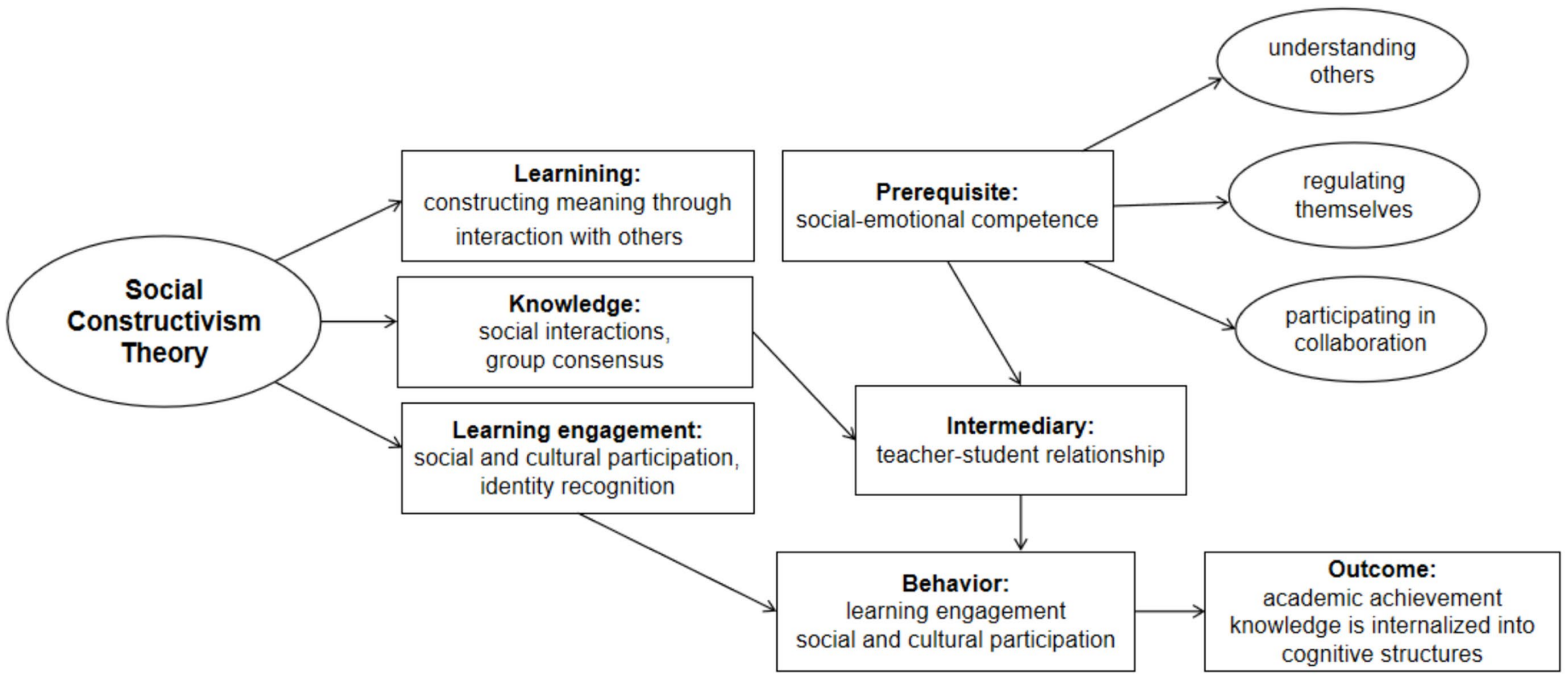
Theoretical framework. Adapted from Berger and Luckmann (1966).

Social Construction Theory indicates that knowledge is not objective fact, but rather constructed through social interactions and agreements between relevant social groups (Berger and Luckmann, 1966). Learning involves constructing meaning through interaction rather than absorbing knowledge in isolation. This approach not only focuses on cognition but also emphasizes the role of emotion and sociality in knowledge construction (Harré, 1986). Students need to possess the abilities of understanding others, regulating themselves and participating in collaboration in order to effectively engage in the social and cultural learning process; and Social Construction theory opposes viewing learning engagement as “individual effort” or “time investment,” but rather as students’ participation and identity in social and cultural practices (Berger and Luckmann, 1966), such as whether they see themselves as “contributors to classroom interaction”. Consequently, this study regards SEC as the prerequisite for students to participate in social and cultural learning, and defines “teacher–student relationship” as a medium for knowledge co-construction; and reconceptualizes “learning engagement as social and cultural participation”, and academic achievement is defined as students effectively transforming external knowledge, skills and values into part of their own cognitive structure through interaction. Figure 1 demonstrates the theoretical framework of this study.

## 4 The present study: hypotheses and proposed model

**Figure 2:**
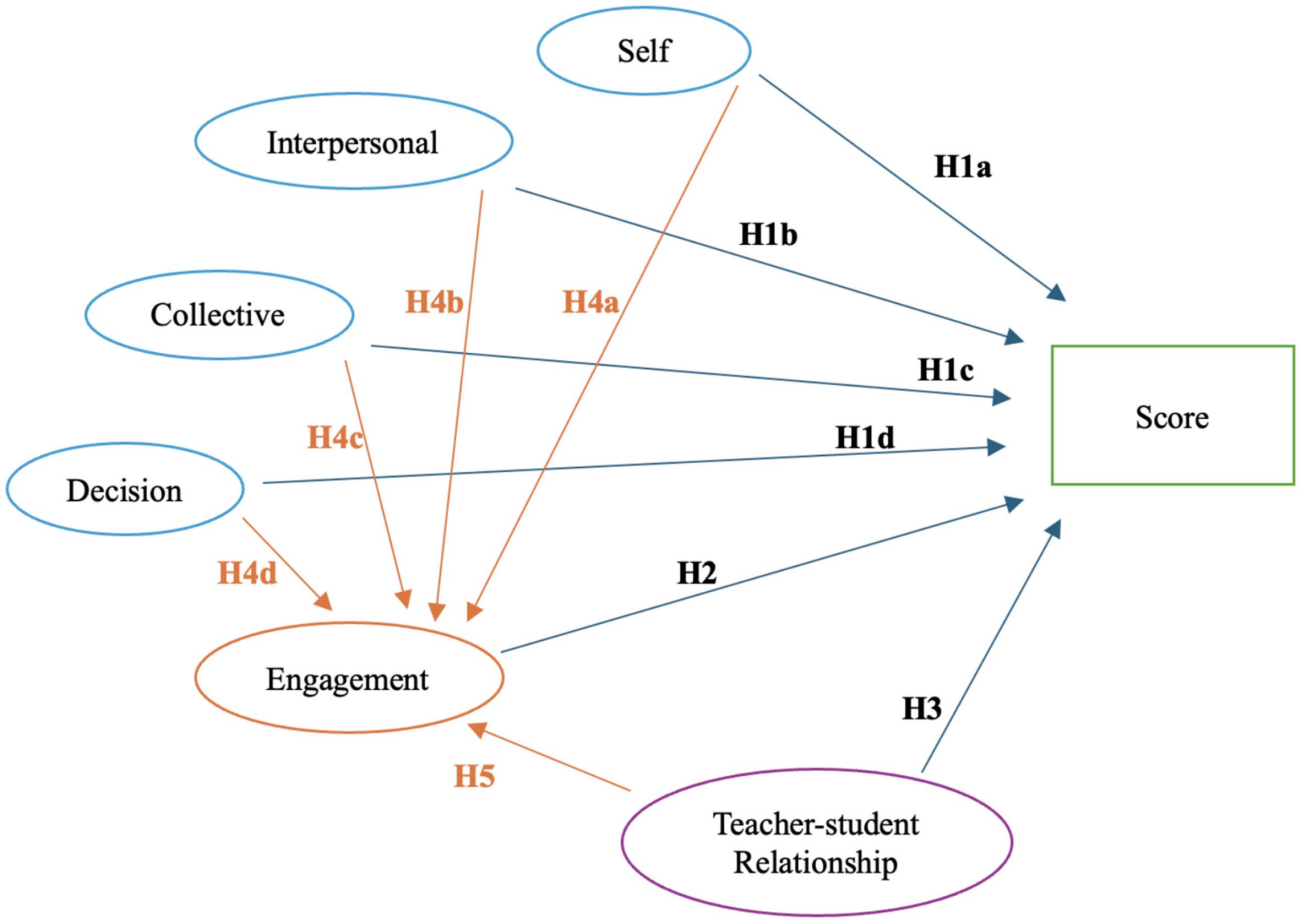
Proposed model.

The study aims to investigate the relationship among social–emotional competence (self-relationship skills, interpersonal relationship skills, collective relationship skills, and responsible decision-making), learning engagement, teacher–student relationship, and academic achievement from the perspective of social construction theory. Figure 2 demonstrates the hypothesized relationships among variables. The proposed hypotheses in the model are as follows:

*H1*: There is a direct positive relationship between social–emotional competence and academic performance, specifically:

*H1a*: There is a direct positive relationship between self-relationship skills and academic achievement.

*H1b*: There is a direct positive relationship between interpersonal relationship skills and academic achievement.

*H1c*: There is a direct positive relationship between collective relationship skills and academic achievement.

*H1d*: There is a direct positive relationship between responsible decision-making and academic achievement.

*H2*: There is a direct positive relationship between learning engagement and academic achievement.

*H3*: There is a direct positive relationship between the teacher–student relationship and academic achievement.

*H4*: There is a direct positive relationship between social–emotional competence and engagement, specifically:

*H4a*: There is a direct positive relationship between the relationship with self and engagement.

*H4b*: There is a direct positive relationship between the interpersonal relationship and engagement.

*H4c*: There is a direct positive relationship between collective relationships and engagement.

*H4d*: There is a direct positive relationship between decision-making and engagement.

*H5*: There is a direct positive relationship between the teacher–student relationship and engagement.

## 5 Method

### 5.1 Participants and procedures

In this study, we adopted purposive sampling to select samples. Therefore, all the samples were from Hunan First Normal University in China. They are students of *the Six-year Normal Education Program* who are taking the course *Education Policy, Regulations, and Teacher Professional Ethics* in the autumn semester of 2024. After graduation, all these students will return to their places of origin to become primary school teachers. Among the 1,528 participants, 28.08% (*n* = 429) are male, and 71.92% (*n* = 1,099) of them are female. For grade, 1.31% (*n* = 20) of them are freshmen, 86.32% (*n* = 1,319) of them are sophomores, 12.17% (*n* = 186) of them are juniors, and 0.20% (*n* = 3) of them are seniors. They are from different majors, including primary education, math, physical education, English, Chinese, music, art, etc. Ethical approval was attained by the Hunan First Normal University. The researcher provided informed consent to the participants, and ethical approval [Number 2024 Ethics (01005)] was obtained from ethical committee at Hunan First Normal University. The approval time was September 9, 2024.

### 5.2 Measures

The researchers conducted a survey among university students using the Social–Emotional Competence Scale, the Teacher–Student Relationship Scale, and the Learning Engagement Scale. Appendix A demonstrated the items of all the measures with the three scales.

#### 5.2.1 Social–Emotional Competence Scale (SECS)

Considering the actual situation of Chinese Students, this article mainly refers to the *Social–Emotional Competence Scale for College Students* made by Chinese scholar Chen et al. (2023) in their research named “*Theoretical model and scale development of university students’ social-emotional competence*”. This scale was revised by referring to Collaborative for Academic, Social, and Emotional Learning (CASEL) (2022) and Organization for Economic Co-operation and Development (OECD) (2021), etc. The social–emotional skills of college students are examined from four dimensions (e.g., self-relationship skills, interpersonal relationship skills, collective relationships, and responsible decision-making skills) with a total of 26 items. Items were measured on a 5-point scale. The higher the score, the higher the level of social emotional ability of college students. The scale has good overall reliability, with a Cronbach’s *α* coefficient of 0.967 (Chen et al., 2023). The Cronbach’s *α* for subscales (self-relationship, interpersonal relationship, collective relationship, and responsible decision making) are 0.873, 0.931, 0.966, 0.923, respectively (Chen et al., 2023).

#### 5.2.2 Teacher–Student Relationship Scale (TSRS)

The Teacher–Student Relationship Scale draws on the scale that is generally applicable to the Chinese context. And this scale was revised by Pu et al. (2024) in their research. This scale measures the teacher–student relationship from two dimensions: communication frequency and communication quality. It consists of a total of seven items including the frequency of communication between teachers and students (Item1) and the teacher–student communication situation (Item 2–7). Items were measured on a 5-point scale. The higher the total score, the better the teacher–student relationship of the student. The scale has good reliability, with a Cronbach’s *α* coefficient of 0.838 (Pu et al., 2024).

#### 5.2.3 Learning Engagement Scale (LES)

The Learning Engagement Scale draws on the scale designed by Symonds et al. (2023), which measured university students’ learning engagement from five aspects. Items were measured on a 5-point scale. Reliability was reasonable with a Cronbach’s *α* coefficient of 0.67 (Symonds et al., 2023).

The Learning Engagement Scale we adapted was originally developed in English and we translated it into Chinese to distribute to participants for better understanding since it is the target population’s native language. In addition, three items that were reverse coded in the original scale were rephrased into positively worded items. Reliability coefficients are sample and context specific, therefore, those language translation, cultural context and item word revisions might contribute to differences in reliability estimate between the prior reported research and this study.

#### 5.2.4 Academic achievement

As the Social Construction Theory emphasizes that academic achievement is when students effectively transform external knowledge, skills and values into their own cognitive structure through interaction, the evaluation of academic achievement should not only include unified and standardized examinations for students at the end of each semester, but also measurements by teachers of students’ regular learning processes. Therefore, academic achievement is mainly based on students’ grades in the course *Education Policy, Regulations, and Teacher.*

The assessment method for this course is the sum of 50% of the regular performance and 50% of the final exam score. Among them, the basis for the assessment of regular performance mainly includes the participants’ classroom performance throughout the semester, attendance rate, course assignments, stage tests, etc. The final exam score is the score of a written closed-book assessment organized in the course, which includes different types of questions. It is worth noting that the regular performance is different from learning engagement. The former mainly refers to the teachers’ assessment of students’ completion of learning tasks and achievement of course objectives. The latter depicts students’ cognitive, emotional and behavioral participation in the learning process, which jointly reflect students’ social and cultural participation/identity recognition.

### 5.3 Data collection and analysis

Questionnaires were distributed online through the *Wenjuanxing* platform at the end of the semester (from December 2024 to January 2025), with the corresponding course instructors providing on-site guidance for filling them out. Then, the sample data were exported from *Wenjuanxing*, and a total of 1,747 sample data were collected. Excluding invalid samples with incomplete or repetitive data filling, a total of 1,528 valid samples were obtained. Cases with substantial missing data were removed using listwise deletion, resulting in an analytic dataset with no remaining missing values. We examined the validity of the instrument through factor analysis, consisting of exploratory factor analysis (EFA) and Confirmatory factor analysis (CFA). Although Likert-type items are ordinal by nature, they were treated as continuous in the SEM analyses, which is common practice when items have five response categories and sample size is large. Accordingly, models were estimated using maximum likelihood (ML), which is robust under these conditions. This estimation method allows for robust assessment of parameter estimates and model fit. Descriptive analysis and correlation analysis were conducted into STATA (18.0). Structural Equation Modeling (SEM) was employed to investigate the model fit and relationship among factors through Mplus software (VERSION 8.1).

## 6 Results

### 6.1 Reliability and validity

This study assesses its appropriateness of Kaiser-Meyer-Olkin measure (KMO = 0.9758), and found outstanding sample adequacy. This shows its strong common variance among items and suitable for factor analysis. Bartlett’s test of sphericity was significant, *χ*^2^(378) = 33386.53, *p* < 0.001, indicate that the assumptions were satisfied and this questionnaire is suitable for exploratory factor analysis (EFA). EFA results suggested eigenvalue greater than 1 are 16 after oblimin oblique rotation. Next, CFA from factor analysis was performed with a specified number one, number three, number four and number six based on theoretical construction.

An exploratory factor analysis (EFA) using principal factor extraction with oblimin rotation was conducted to examine the underlying structure of the scale. A one-factor solution accounted for 74.69% of the common variance (eigenvalue = 18.40), with all items loading positively (0.50–0.80); however, several items exhibited high uniqueness, indicating that a single factor did not adequately capture all systematic variance. This was further supported by a one-factor confirmatory factor analysis (CFA), which demonstrated poor model fit [*χ*^2^(665) = 15,405.18, *p* < 0.001; RMSEA = 0.120; CFI = 0.695; SRMR = 0.091], suggesting that the covariance structure could not be explained by a single latent construct and that common method variance was unlikely to dominate the observed relationships. A three-factor EFA solution yielded factor loadings exceeding 0.50; however, engagement items failed to load cleanly on a distinct factor and instead exhibited cross-loadings across self-focused social–emotional competence, other-focused social–emotional competence, and teacher–student relationship. Consequently, a four-factor solution was examined, which produced more clearly defined factors; nevertheless, engagement items continued to show moderate cross-loadings and relatively low factor loadings (0.31–0.44), and several other-focused social–emotional competence items (Items 20–23) loaded onto other factors. The four-factor model also demonstrated suboptimal fit [*χ*^2^(659) = 8,392.65, *p* < 0.001; RMSEA = 0.088; CFI = 0.840; SRMR = 0.073].

We conducted CFA using six factor solution, which meets the theoretical framework and statistical requirements. The eigenvalues of all six factors were greater than 1. The items loaded clearly on 6 factors with standardized factor loading ranging from 0.39 to 0.87 (see Table 1). All items factor loadings are greater than 0.50 except for six items, however, the uniqueness is not greater than 0.60 and there is no serious cross-loading problem or conceptual mismatch. After comparing the alternative models, the six-factor solution was determined to provide the best fit.

**Table 1:** Standardized CFA solution.

Items	Factor loading					
	Responsible decision-making	Collective	Learning engagement	Teacher–student	Interpersonal	Self
Item 1						0.41
Item 2						0.68
Item 3						0.59
Item 4						0.63
Item 5						0.71
Item 6						0.68
Item 7					0.49	
Item 8					0.75	
Item 9					0.70	
Item 10					0.77	
Item 11					0.54	
Item 12		0.56				
Item 13		0.80				
Item 14		0.84				
Item 15		0.46				
Item 16		0.77				
Item 17		0.74				
Item 18		0.43				
Item 19		0.51				
Item 20	0.61					
Item 21	0.60					
Item 22	0.66					
Item 23	0.39					
Item 24	0.71					
Item 25	0.67					
Item 26	0.58					
Item 27				0.80		
Item 28				0.80		
Item 29				0.73		
Item 30				0.87		
Item 31				0.83		
Item 32				0.58		
Item 33				0.66		
Item 34			0.41			
Item 35			0.62			
Item 36			0.67			
Item 37			0.71			
Item 38			0.60			
Eigenvalue	13.64	13.41	11.74	10.82	9.74	9.19

Convergent and discriminant validity were examined to evaluate construct validity. To establish convergent validity, standardized factor loadings are expected to exceed 0.50. However, items with loadings slightly lower than 0.50 may be retained when the average variance extracted (AVE) exceeds 0.50. It is recommended that the AVE from each construct is equal to or higher than 0.50 and less than Cronbach alpha and composite reliability values (Fornell and Larcker, 1981; Hair et al., 2010). Table 2 suggested that composite reliability and Cronbach’s alpha values are larger than AEV and all six constructs demonstrated AVE values above 0.50, supporting adequate convergent validity. The Cronbach’s alpha values for six constructs are all over 0.70 suggesting sufficient reliability.

Discriminant validity is the degree of one construct distinguished from another. Discriminant validity is supported when the square root of each construct’s AVE is greater than its correlations with the remaining constructs (Fornell and Larcker, 1981; Hair et al., 2010). Table 2 demonstrated the square root of AVE values are larger than the correlations, supporting discriminant validity.

**Table 2:** The average variance values and the reliability coefficients.

Measurement index	Self relationship	Interpersonal relationship	Collective relationship	Responsible decision making	Learning engagement	Teacher–student relationship
Average variance extracted	0.5739	0.7631	0.6845	0.6275	0.5585	0.6535
Composite reliability	0.8890	0.9279	0.9455	0.9213	0.8633	0.9294
Cronbach’s alpha	0.8861	0.9259	0.9448	0.9145	0.8606	0.9269

Table 3 demonstrates the descriptive statistics of the instruments after deleting item 7. Student exam score was used as academic achievement (Mean = 79.83, SD = 7.43, Min = 31, Max = 96). The modified items reached the reliability of *a* = 0.9696 with 37 remaining items. Table 4 demonstrates the correlations among variables.

**Table 3:** Descriptive statistics of variables (*n* = 1,528).

Descriptive statistics	Self relationship	Interpersonal relationship	Collective relationship	Responsible decision making	Learning engagement	Teacher–student relationship
Mean	22.71	17.42	33.62	28.70	19.72	26.11
SD	4.12	2.61	5.20	4.42	3.52	5.37
Min	6	4	8	7	5	7
Max	30	20	40	35	25	35

**Table 4:** Results of discriminate validity and correlation among variables.

Variable	1	2	3	4	5	6
1	0.7589					
2	0.53^***^	0.8736				
3	0.62^***^	0.77^***^	0.8273			
4	0.69^***^	0.71^***^	0.79^***^	0.7922		
5	0.64^***^	0.53^***^	0.66^***^	0.71^***^	0.7473	
6	0.60^***^	0.41^***^	0.55^***^	0.58^***^	0.69^***^	0.8084
Score	0.05^*^	0.10^***^	0.13^***^	0.06^*^	0.08^*^	0.05^*^

1 = self-relationship, 2 = interpersonal relationship, 3 = collective relationship, 4 = responsible decision making, 5 = engagement, 6 = teacher–student relationship.

The values on the diagonal are the square root of AVE values. The values below the diagonal are the correlations. ^*^p < 0.05, ^***^p < 0.001.

### 6.2 Measurement model

We included six latent variables in the measurement model (*self-relationship skills, interpersonal relationship skills, collective relationship skills, responsible decision making, learning engagement, teacher–student relationship*). Among those, self-relationship skills, interpersonal relationship skills, collective relationship skills, and responsible decision making are four dimensions of social–emotional competence. Initially, the factor loading ranged from 0.40 to 0.87 and loaded on six distinct factors. Item 7 was supposed to be loaded on construct of self-relationship from the original adopted survey; however, it loaded on construct of interpersonal. We examined Item 7 (I respect myself and hope to be respected by others) conceptually and found that it mentioned both self and interpersonal relationship components, so we deleted this item due to cover both construct conceptually and potential a cross-loading issue.

For the measurement model, we used the following criteria for fit indices, including the root mean square error of approximation (RMSEA) with a threshold value of 0.08, standardized root mean squared residuals (SRMR) with a threshold value of 0.08, and the comparative fit index (CFI) with a threshold value of 0.90 (Byrne, 1994). The measurement model reached an acceptable fit for the measurement model [*χ*^2^ (614) = 4517.726, *p* < 0.001, RMSEA = 0.065, CFI = 0.917, SRMR = 0.054].

### 6.3 Structural model

A Structural Equation Modeling (SEM) was constructed with self-relationship skills, interpersonal relationship skills, collective relationship skills, responsible decision making, and teacher–student relationship and learning engagement as the six latent variables and score as the observed variable. Exogenous variables are self-relationship skills, interpersonal relationship skills, collective relationship skills, responsible decision making, and the teacher–student relationship. Endogenous variables are engagement and score.

Table 5 demonstrates the results of the structural model. The model demonstrated a good fit [*χ*^2^ (645) = 4594.116, *p* < 0.001, RMSEA = 0.063, CFI = 0.916, SRMR = 0.053]. Notably, we discovered a significant relationship among the dimensions of social–emotional competence and academic achievement. Specifically, a positive relationship between collective relationship and academic achievement (*β* = 0.20, *p* < 0.05) and a significant relationship between responsible decision making and academic achievement (*β* = −0.16, *p* < 0.05). Regarding factors affecting learning engagement, we found a significant relationship between all dimensions of social emotional competence and engagement. Namely, self-relationship and learning engagement (*β* = 0.14, *p* < 0.001), interpersonal and learning engagement (*β* = −0.07, *p* < 0.05), collective/group relationship and learning engagement (*β* = 0.18, *p* < 0.001), responsible decision making (*β* = 0.34, *p* < 0.001), and a significant relationship between teacher–student relationship and engagement (*β* = 0.38, *p* < 0.001).

**Table 5:** Model results.

Hypothesis	Direct effect	*β*	*p*
*H1a*	Self → academic achievement	−0.02	0.68
*H1b*	Interpersonal → academic achievement	0.05	0.35
*H1c*	Collective → academic achievement	0.20	<0.05^*^
*H1d*	Decision-making → academic achievement	−0.16	<0.05^*^
*H2*	Engagement → academic achievement	0.10	0.16
*H3*	Teacher student relationship → academic achievement	−0.04	0.40
*H4a*	Self → engagement	0.14	<0.001^***^
*H4b*	Interpersonal → engagement	−0.07	<0.05^*^
*H4c*	Collective → engagement	0.18	<0.001^***^
*H4d*	Decision making → engagement	0.34	<0.001^***^
*H5*	Teacher–student relationship → engagement	0.38	<0.001^***^

^*^p < 0.05, ^***^p < 0.001.

**Figure 3:**
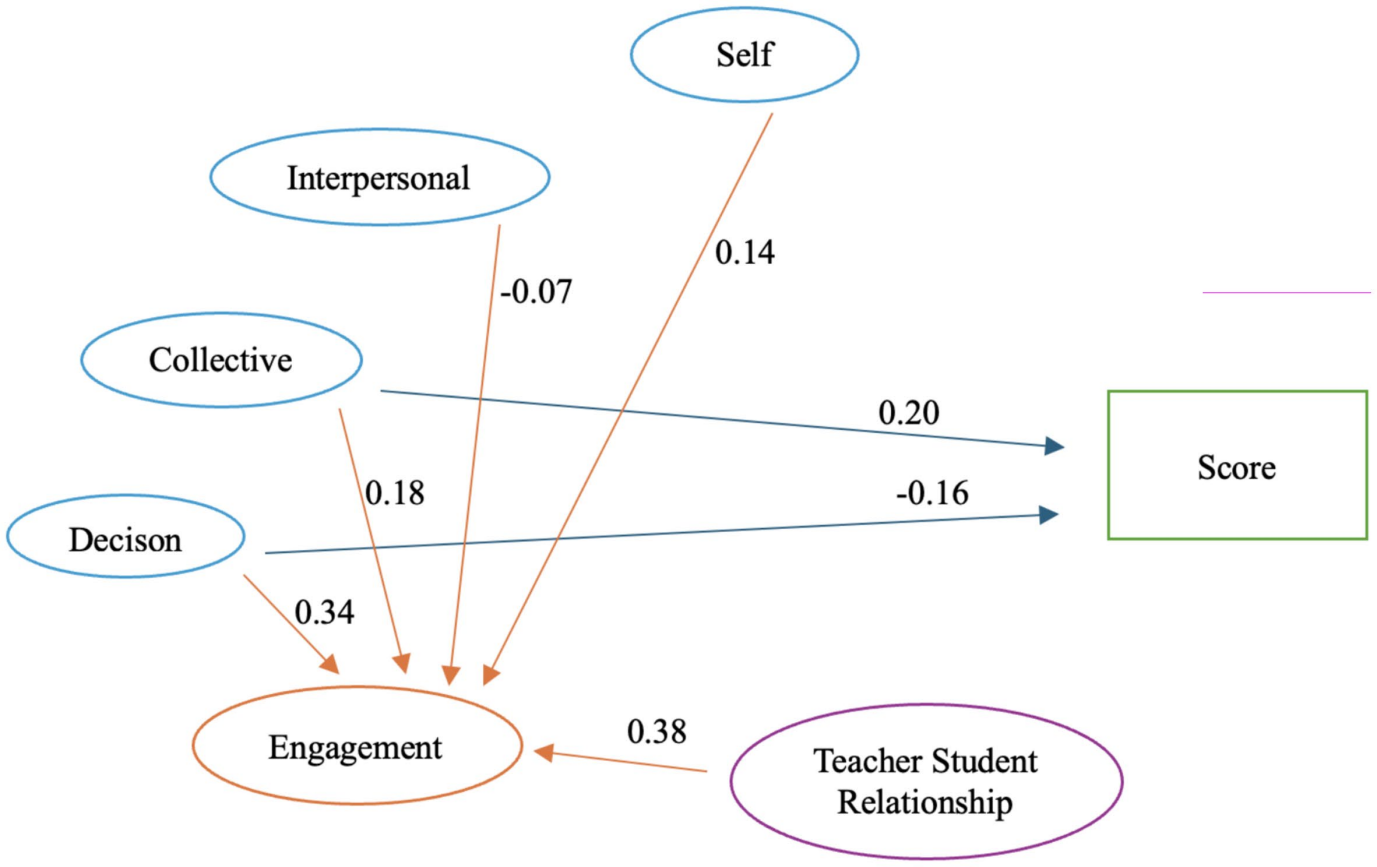
Path diagram of the structure model with significant relationships.

As seen in Table 5, *H1c*, *H4a*, *H4c*, *H4d*, *H5* were supported but *H1a*, *H1b*, *H2*, *H3* were not supported. *H1d* and *H4b* were partially supported since with the significant prediction/relationship but the coefficients were found to be negative, so the direction of the relationship was not supported. The negative coefficients here should be interpreted with caution. In Table 4 there was no negative correlation detected among the variables. The negative path coefficients might be as conditional (suppression) effects reflecting shared variance among related SEC dimensions rather than substantive true negative associations. Figure 3 demonstrates the model results with significant relationships.

## 7 Discussion

### 7.1 Collective relationship skills and academic achievement

According to previous research, SECs are not only closely related to students’ academic achievement (Coneus and Laucht, 2014; Portela-Pino et al., 2021) but also have a profound impact on their emotion regulation (Chen L. et al., 2024). We found that collective relationship skills, which refer to the ability to handle relationships with the group, positively predict students’ academic achievement. This finding implies that it is not sufficient for current teacher education only target knowledge acquisition, while team-based collaborative learning is likely as important, which is supported by Social Construction Theory. This theory emphasizes that learning is essentially not the one-way transmission of information, but rather the construction of meaning achieved through interaction and collaboration within a social network (Berger and Luckmann, 1966). The previous research has confirmed that collective relationship competence significantly and negatively predicts psychological anxiety (Chen and Chen, 2025), it indicates that collective relationship skills can help students relieve learning anxiety and thereby promote their improvement in learning performance. It can also be inferred that students with strong collective relationship skills tend to integrate into learning communities such as classes and clubs more quickly, forming stable learning mutual assistance circles. For instance, in cooperative tasks, they can efficiently coordinate and divide tasks, allowing each member to focus on their areas of expertise and avoid repetitive work, thus enabling all the group members to grow together.

In this case, educators should consider cultivating the pre-service teachers’ collective relationship skills during their learning process, instead of simply focusing on knowledge acquisition or pedagogical skills training. This also depends on the diversified reform and optimization of the evaluation system for teacher education curriculum. A unified written test at the end of the term has its limitations; instead, we recommend combining it with process-oriented evaluations that include collaborative learning, teamwork and interpersonal relationships, so as to promote student’ cultural participation in social relations.

### 7.2 Responsible decision-making and academic achievement

Responsibility, as the core of ethics, is an important bond for maintaining social relations and a key component of moral education (Durkheim, 1961). According to Osher et al. (2016), responsible decision-making (RDM) refers to the capacity to make choices based on realistic evaluations of consequences, well-being, ethics, safety, and social norms. According to the CASEL framework, developing RDM competence is considered one of the key means for promoting individual well-being [Collaborative for Academic, Social, and Emotional Learning (CASEL), 2022].

RDM positively impacts the learners’ performance, interpersonal and intrapersonal relationships, and emotional competencies in their studies (González-Sanz and Pichardo, 2018; Portela-Pino et al., 2021; Weissberg et al., 2015). Our study did not find that positive association due to possible conditional (suppression) effects. Previous studies have suggested that RDM necessitates considering ethical principles, safety concerns, societal norms, and the potential consequences of one’s actions on oneself, others, and the broader community (Najjarpour and Salimi, 2024). Our study did not confirm the positive association between RDM and academic performance, future research are encouraged to investigate the direction of the correlation.

RDM is one of the competencies of SECs, which are well established as critical skills for healthy and adaptive youth development (Santos et al., 2023). Consequently, the evaluation mechanism of higher education should be designed to consider those students who can think twice before acting when facing problems and challenges, consider the pros and cons of their actions, and take responsibility for themselves, others, teams, or collectives, instead of taking examination performance as the core and only goal. Moreover, the current university curriculum system should be optimized. Therefore, the formal courses should be included instead of traditional moral lectures to enhance students’ attention and engagement. For instance, set up courses covering data ethics, AI plagiarism, etc., to promote the transfer of students’ RDM ability to academic behavior, thereby improving it simultaneously with their academic achievements.

### 7.3 SECs and learning engagement

Although few studies explored how the specific components of SECs influence the students’ engagement, it has been verified that SECs have positive relevant correlations with learners’ engagement (Santos et al., 2023). Hence, SECs can generally promote a students’ engagement. In this study, we analyzed the relationships between the four different dimensions and learning engagement through Structural Equation Modeling (SEM). The results indicated that self-relationship skills, collective relationship skills, and RDM skills of students are positively associated with their learning engagement, while interpersonal relationship skills is linked to their learning engagement. This finding supports the view of Social Constructivism that cognition, emotion and sociality are regarded as an inseparable whole (Berger and Luckmann, 1966), and students with higher SECs are more likely to actively participate in meaningful negotiation in group cooperation and classroom interaction. This kind of participation is precisely the learning mechanism emphasized by Social Constructivism.

As the important contents of SECs, self-relationship skills, collective relationship skills, and RDM skills is positively correlated with students’ engagement, which is consistent with the prior research in general. Self-relationship refers to the ability that students have to handle the relationship with themselves, and the earlier research revealed that self-management skills are closely related to maladjustment, school readiness, and school participation (Eisenberg et al., 2010). Collective relationship competence (CRC) represents a unique dimension within the SEC framework of Chinese students, which enables students to function effectively in group settings, manage individual-group dynamics, and adhere to collective values and behavioral norms (Chen and Chen, 2025). It is confirmed that college students’ collective relationship is significantly important for reducing their psychological anxiety and the likelihood of mental health issues, and supporting the maintenance or improvement of mental health (Chen and Chen, 2025). While some previous studies have verified that anxiety can negatively influence students’ learning engagement (Lizarte Simón et al., 2024; Mou et al., 2022; Ng et al., 2022). In this situation, collective relationship skills can promote the improvement of students’ meaningful learning engagement.

Responsible decision-making, the key dimension of SECs, highlights the importance of considering not only personal benefits and risks but also those of others when making decisions, fostering thoughtful decision-making and preventing impulsive choices through an awareness of one’s impact on others (Mahoney et al., 2021). Our result indicates that RDM is positively associated with students’ engagement, which is consistent with the viewpoints of prior studies [Chen Z. et al., 2024; Collaborative for Academic Social and Emotional Learning (CASEL), 2003; Santos et al., 2023]. RDM is the result of college students’ learning engagement and will be strengthened as the degree of learning engagement increases. Conversely, RDM will also help college students better handle their relationships with peers, teachers, and the school collective, thereby enhancing the effectiveness of learning engagement (Chen Z. et al., 2024). Therefore, RDM is an important factor that is associated with pre-service teachers’ engagement.

Interpersonal relationships affect the ability to handle relationships with others. According to Collie et al. (2016), students’ interpersonal relationships with teachers, parents, and peers are positively associated with academic engagement. Our study indicates that interpersonal relationships are relevant to learning engagement. Although the coefficient is negative, it does not necessarily mean this is true negative association. As shown in Table 3, all the correlation among variables are positive. The result of this negative coefficient from the SEM should be interpreted with caution duet to possible conditional (suppression) effects reflecting shared variance among related SEC dimensions rather than substantive true negative associations. Our study did not confirm a positive direction of interpersonal relationship and academic performance; future studies are encouraged to further investigate this.

From the relations that we analyzed, specific SECs between students’ engagement, some recommendations are raised to reform the current situation of teacher education. The cultivation of students’ SECs should be incorporated into the curriculum system of teacher education, which is crucial for pre-service teachers’ development and academic achievements. Otherwise, it is not the case that the higher a students’ SECs are, the better. Educators should pay attention to the degree of pre-service teachers’ interpersonal relationships, avoiding their overly high interpersonal skills from reducing their learning engagement instead.

### 7.4 Teacher–student relationship and learning engagement

The teacher–student relationship is an important relationship for students to participate in educational and teaching activities, which directly affects their learning outcomes. Our research found it closely with pre-service teachers’ learning engagement, which is consistent with the previous studies (He and Li, 2024; Li, 2023; Wang and Yuan, 2024). This finding is also largely consistent with Social Construction Theory. According to Vygotsky’s concept of the *Zone of proximal development,* it states that learning occurs in the gap between the “actual level of development” and “the potential level of development”, with this gap being achieved through adult guidance or by collaborating with more capable peers (Vygotsky, 1978). This study indicates that a positive teacher–student relationship precisely creates an ideal cooperative environment where teachers, as experts, build cognitive scaffolds for students, reducing cognitive uncertainty. Hence, the teacher–student relationship promotes students’ identity construction, knowledge construction, meaning construction, and social and cultural participation.

Consequently, the quality of the teacher–student relationship should be included in the assessment indicators of teacher education to enhance instructors’ motivation to improve their relationship with students. In addition, teacher preparation universities should implement small-class teaching, even for public courses, ensuring that every student is seen by the teacher so students can fully participate in this learning community. Especially with the widespread use of AI and the trend of human-computer dialogue, the importance of the teacher–student relationship should be given even more attention. Research conducted by Liu and Peng (2025) reveals that while AI has significant advantages in enhancing educational efficiency, it has limitations in interpersonal emotional support and the transformation of the teachers’ role. As a result, educators should enhance emotional care and improve students’ emotional literacy to ensure the comprehensiveness, validity, and efficiency of higher education. Besides, the notion among teachers that the significant role of interaction in the co-construction of knowledge (Mercer, 2000), should be strengthened. Hence, in the classroom, educators are more often regarded as guides of students’ learning, providing an environment where students are able to express their own opinions and helping them construct new knowledge.

Moreover, the teachers’ relational literacy should be more considered and cultivated. Thus, care can be done professionally rather than casually. For example, universities with teacher preparation programs can set up micro-workshops on *Communication of Relations among College Teachers.*

## 8 Conclusion, limitations, and future directions

### 8.1 Conclusion

In the current context where digital technology is increasingly integrated into education, SECs, teacher–student relationships, and learning engagement are important factors influencing students’ academic achievement. This study explored how the dimensions of SECs, learning engagement, and teacher–student relationships impact students’ academic achievement from the perspective of Social Construction Theory. The study reveals that there are direct positive relationships between collective relationship skills and academic achievement, which are consistent with the prior research findings that SECs positively influence academic achievement (Rhoades et al., 2011; Wirajaya et al., 2019; Zahid et al., 2024). A direct relationship between RDM and academic achievement. The study also found that SECs and the teacher–student relationship is linked to students’ engagement. For example, collective relationship and RDM of students impact their learning engagement, which is also consistent with the previous findings (Chen Z. et al., 2024; Santos et al., 2023; Yang, 2025). Although the coefficient of the correlation of RDM and academic achievement and the correlation coefficient between the interpersonal relationship and engagement are negative, this might not reflect a true negative association due to possible conditional (suppression) effects in the model.

These viewpoints that differ from the previous studies may stem from the fact that the distinction of the survey samples. This study investigated pre-service teachers in universities. Meanwhile, these different research conclusions have also provided us with new inspirations and thoughts on current teacher education and higher education. Research has shown that the current higher education and teacher education system does not merely take students’ test-taking ability as the educational goal. However, the evaluation system for students who are trained to be future teachers still needs to be reformed in the point of social construction. In particular, the diversification of evaluation indicators, contents, and methods for pre-service teachers should be strengthened to avoid neglecting the recognition of their responsible decision-making ability. This study also indicates that in the current situation where big data and AI are widely applied, where the curriculum system of teacher education should not only attach importance to the cultivation of students’ professional abilities and teaching skills, but also pay special attention to the cultivation of students’ moral qualities. In addition, it is vital to attach great importance to the cultivation of the teacher–student relationship among pre-service teachers, strengthen the development of teachers’ relationship skills, and ensure that pre-service teachers receive sufficient emotional support, respect, trust, and care.

### 8.2 Limitations and future directions

Although this study analyzed the interaction mechanism among SECs, the teacher–student relationship, learning engagement, and academic achievement through SEM, it reached feasible and somewhat innovative viewpoints and conclusions. However, this study also has certain limitations as follows:

First, the representativeness of the sample is insufficient. Although the sample size of this study is over a thousand, since the study selected students from a certain normal university as the research subjects, the diversity of the sample is inadequate, which may lead to the research conclusion having the characteristics of a certain college, thus limiting its generalizability. Future studies are encouraged to collect samples from normal colleges and universities across the country rather than being limited to a single institution, to enhance the diversity and representativeness of the samples, thereby improving the reliability and scientific nature of the results.

Second, this study did not explore the mediating and moderating effects among the variables. Previous studies have found that the influence of SECs on students’ academic achievement is exerted through teacher–student relationships, student–student relationships, etc. This study did not conduct explorations in these aspects. However, the interaction mechanism between SECs in different dimensions, learning engagement, teacher–student relationship, and academic achievements of pre-service teachers is still worthy of further in-depth research. Researchers can further explore the advancement of the global educational digitalization building on social constructivism.

Third, we all used self-report measures to assess SECs, learning engagement, and teacher–student relationships. Although self-reporting is especially beneficial in the assessment of self-relationship dimensions of SECs since they cannot be observed directly, future research should explore other measures, maybe more objective observations of SECs, learning engagement, and the teacher–student relationship. In particular, interactive discourse analysis should be used to measure learning engagement. At the same time, there might be some overlaps between learning engagement and regular performance since the attendance used in the regular performance can be considered as engagement, and future research may consider modeling academic achievement based on examinations only. In addition, given the cross-sectional, self-report design, causal interpretations cannot be drawn. Although steps were taken to mitigate this risk, including the use of established and psychometrically validated scales, careful item wording, and assurances of respondent anonymity, the presence of common method bias cannot be entirely ruled out. Future research using longitudinal or multi-source data or experimental designs would strengthen causal inference.

## Data Availability

The raw data supporting the conclusions of this article will be made available by the authors, without undue reservation.
