# Supplementary material for: Linking social–emotional competence, learning engagement, and teacher–student relationship to academic achievement: a structural equation model approach
Source: Front Psychol. 2026 Mar 16;17:1756766. doi: 10.3389/fpsyg.2026.1756766 (PMC13033739; doi:10.3389/fpsyg.2026.1756766)
Supplement: Supplementary file 1 [file Data_Sheet_1.pdf]

Appendix A

Social-Emotional Competence Scale		
Constructs	Items	N
Self-relationship		7
	1. I know my strengths and weaknesses	
	2. I believe I have the ability to complete the task	
	3. I can regulate my emotions and express them in an appropriate way	
	4. I can reflect on myself and make improvements	
	5. I can overcome difficulties and persist in completing tasks	
	6. I can set long-term goals and will strive for them until they are achieved	
	7. I respect myself and also hope to be respected by others.	
Interpersonal relationship		4
	8. I know to be kind to others	
	9. I know I need to put myself in others' shoes and sympathize with their feelings	
	10. I know that I should respect others' thoughts, emotions and beliefs	
	11. I can understand and tolerate others' thoughts, emotions and behaviors.	
Collective relationship		8
	12. I can cooperate with others to complete common collective tasks	
	13. I know that a sense of collective belonging is very important	

	14. I identify with collective values and collective behavioral norms	
	15. I know that everyone in the collective is equal and important	
	16. I actively integrate into the collective and abide by the collective rules	
	17. I can safeguard the collective honor and actively contribute to it	
	18. In a collective, I can distinguish between my own and others' responsibilities	
	19. I strive to maintain a harmonious atmosphere for collective study and life.	
Responsible decision-making		7
	20. I always think twice before I act in dealing with people and matters	
	21. I can be responsible for my actions; 22) I can judge the right and wrong of things	
	22. I can judge the right and wrong of things	
	23. When I'm in trouble, I won't blame fate or others	
	24. The decisions I make are not only responsible for myself, but also for others and the collective	
	25. I can weigh the impact of the decisions I make on myself, others and the collective interests, and make appropriate adjustments when necessary	
	26. When making choices or decisions, I can take into account social security and moral norms	
Teacher-student Relationship Scale		
Constructs	Items	N
Teacher-student Relationship		7
	27. I often have heart-to-heart exchanges with my teachers	

	28. When I encounter difficult problems, I am willing to seek the teacher's help	
	29. I am willing to be close to my teacher	
	30. I am often cared for and encouraged by my teachers	
	31. My creative insights in study are often affirmed by my teachers	
	32. The teacher will give us the opportunity to fully express our opinions	
	33. I have a harmonious relationship with my teacher	
Learning Engagement Scale		
Constructs	Items	N
Learning Engagement		5
	34. Always willing to help the teacher	
	35. I like school	
	36. Feel school is not largely a waste of time	
	37. Quiet in the classroom and get on with work	
	38. Find it not difficult to keep their mind on work	
